# In the midst of a pandemic, more introverted individuals may have a mortality advantage

**DOI:** 10.1016/j.dialog.2022.100087

**Published:** 2022-11-30

**Authors:** Dana A. Glei, Maxine Weinstein

**Affiliations:** Center for Population and Health, Georgetown University, 312 Healy Hall, 37^th^ and O Streets, NW, Washington, DC 20057-1197, USA

**Keywords:** Mortality, Extroversion, Introversion, COVID-19, Pandemic, United States

## Abstract

**Purpose:**

We investigated whether the relationship between extroversion and mortality changed during the COVID-19 pandemic.

**Methods:**

Midlife Americans were surveyed in 1995–96 with mortality follow-up through December 31, 2020. We used a Cox model to estimate age-specific mortality controlling for sex, race/ethnicity, the period trend in mortality, an indicator for the pandemic period (Mar-Dec 2020), extroversion, and an interaction between extroversion and the pandemic indicator.

**Results:**

Prior to the pandemic, extroversion was associated with somewhat lower mortality (HR = 0.93 per SD, 95% CI 0.88–0.97), but the relationship reversed during the pandemic. Extroversion was associated with greater pandemic-related excess mortality (HR = 1.29 per SD, 95% CI 1.002–1.67). That is, compared with persons who were more introverted, those who were highly extroverted suffered a bigger increase in mortality during the pandemic relative to pre-pandemic mortality levels.

**Conclusions:**

The slight mortality advantage enjoyed by more extroverted Americans prior to the pandemic disappeared during the first 10 months of the COVID-19 pandemic. We suspect that the mortality benefit of introversion during the pandemic is largely a result of reduced exposure to the risk of infection, but it may also derive in part from the ability of more introverted individuals to adapt more easily to reduced social interaction without engaging in self-destructive behavior (e.g., drug and alcohol abuse).

## Graphical abstract

**Figure:**
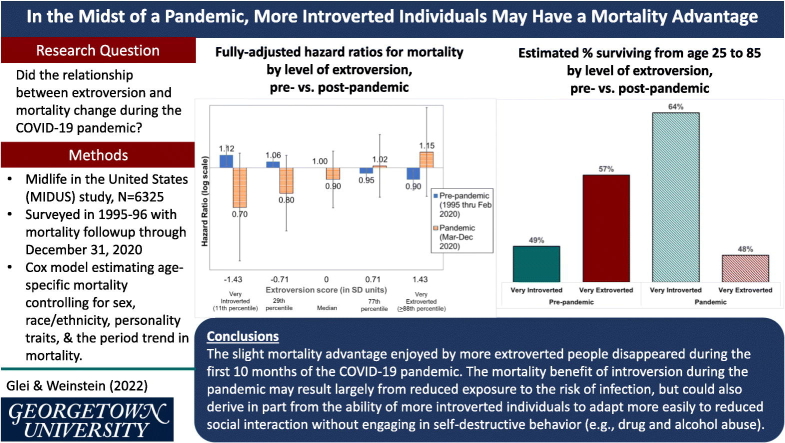

## 1 Introduction

Under normal circumstances, people who are more extroverted (i.e., those energized by social situations [1]) may enjoy lower mortality than more introverted individuals (i.e., who prefer less socially stimulating environments [1]) [[2], [3], [4]]. The health-behavior model of personality suggests that the main mechanism through which personality affects mortality is by influencing one's propensity to adopt health-promoting behaviors while avoiding harmful behaviors [5,6]. For example, extroversion could improve survival by enhancing social relationships [[7], [8], [9]].

Yet, extroversion could become a detriment during an airborne pandemic that thrives on human contact. Social life changed abruptly when COVID-19 was declared a global pandemic. In the interest of reducing contagion, social interaction was severely curtailed when offices, non-essential businesses, and schools were closed, while large social gatherings were canceled. We suspected that individuals who were more introverted were more willing to limit social activities and avoid large gatherings of people, thereby lowering risk of exposure to SARS-CoV-2. Apart from exposure-to-risk, we thought people who were more introverted may have been better-equipped to cope with reduced social interaction while maintaining healthy behaviors without resorting to risk-taking behavior such as substance abuse. If so, we hypothesized that more introverted people would have experienced fewer adverse consequences of the pandemic than their more extroverted counterparts.

To our knowledge, no one has evaluated the effect of extroversion on excess mortality during the pandemic, although there has been considerable attention paid to subjective feelings of loneliness, anxiety, and depression. Most of those studies suggest that extroversion was associated with bigger increases in loneliness [10,11] and greater deterioration in mental health during the pandemic [12,13]. Similarly, we expected that more extroverted individuals experienced more excess mortality during the pandemic than those who were more introverted.

In this paper, we use data for midlife Americans surveyed in 1995–96 with mortality follow-up through December 31, 2020 to investigate whether the association between extroversion and mortality in the US changed during the COVID-19 pandemic. A priori, we hypothesized that there would be an inverse association between extroversion and mortality during the pre-pandemic period (1995-Feb 2020), but the relationship would be reversed during the pandemic (March-Dec 2020). That is, we anticipated that excess mortality during the pandemic would be greater for individuals who were more extroverted than for those who were highly introverted.

## 2 Materials and methods

The data came from the Midlife in the United States (MIDUS) study, which surveyed Americans in 1995–96 with mortality follow-up through December 31, 2020 (see Appendix A for details). Among the 6325 respondents who completed the mail-in self-administered questionnaires at baseline, 1767 (27.9%) died by December 31, 2020.

Personality was measured at Wave 1 using the standardized questionnaire for the “Big Five” taxonomy of personality [14]. We included potential confounders that may affect personality and are known to be associated with mortality: sex, age, and race/ethnicity. Table S1 shows descriptive statistics for all analysis variables.

We used standard practices of multiple imputation to handle missing data [15,16]. A Cox model was used to model age-specific mortality with a robust variance estimator to correct for family-level clustering. Model 1 adjusted for age (as the time metric), sex, race/ethnicity, calendar year (i.e., to capture period mortality decline), a dichotomous indicator (*P*) for the pandemic period, extroversion (*E*), and an interaction between the extroversion score and the pandemic indicator.

The pandemic indicator represents the extent to which mortality after March 2020 differed from the expected level of mortality in the absence of a pandemic after accounting for cohort aging and period mortality decline. A hazard ratio (HR) greater than 1.0 implies excess mortality during the pandemic (i.e., mortality was higher than expected based on the pre-pandemic mortality linear trend), whereas a value less than 1.0 indicates that mortality was lower than expected. Excess mortality includes deaths resulting directly from COVID-19 (whether recorded as such or not) as well as potential increases in mortality from other causes indirectly affected by the pandemic.

To ease interpretation, we reparameterized the model to include two interaction effects for extroversion rather than a main and an interaction effect. The first interaction (*E* × (1 − *P*)) represents the effect of extroversion during the pre-pandemic period; it is the same as the main effect in a standard specification. For this interaction, we expected a hazard ratio less than 1.0, indicating that individuals who scored higher on extroversion experienced lower mortality prior to the pandemic than those who were more introverted. The second interaction (*E* × *P*) represents the effect of extroversion during the pandemic period; the coefficient for this interaction equals the sum of the main effect and the interaction effect from the standard specification. For this interaction, we expected a hazard ratio greater than 1.0, implying that those who scored higher on extroversion experienced higher mortality during the pandemic than their more introverted counterparts.

Model 2 further adjusts for the main effect of conscientiousness, which is the personality trait previously reported to be most strongly and consistently associated with mortality [2,3,[17], [18], [19]]. We might expect conscientious individuals to exhibit greater compliance with public health orders to socially distance, wear a mask in higher-risk settings, accept vaccination, and stay up-to-date with appropriate boosters. As expected, conscientiousness conferred a mortality advantage even before the pandemic. In auxiliary models, we tested an interaction between conscientiousness and the pandemic indicator, but found no evidence that the effect of conscientiousness differed significantly between the pre-pandemic and pandemic period. That is, conscientiousness continued to be associated with lower mortality throughout the period, but there was no indication that the mortality advantage increased during the pandemic. In Model 3, we added the other three personality traits (i.e., neuroticism, openness, and agreeableness).

## 3 Results

Prior to the pandemic, extroversion was associated with somewhat lower mortality (HR = 0.93 per SD, 95% CI 0.88–0.97; Table S2, Model 1). In contrast, the effect of extroversion reversed during the pandemic: more extroverted individuals appeared to suffer higher mortality than their introverted counterparts, although the effect was not significant (HR = 1.20 per SD, 95% CI 0.93–1.54). Given the relatively small number of deaths during March–December 2020 (*N* = 79, 14 of which were reported to have resulted from COVID-19), the confidence intervals are very wide for the pandemic period. Nonetheless, the results indicate that extroversion was associated with greater pandemic-related excess mortality (HR = 1.20/0.93 = 1.29 per SD, 95% CI 1.002–1.67); that is, compared with those who scored higher on introversion, people who were more extroverted suffered a bigger increase in mortality during the pandemic relative to their pre-pandemic mortality levels.

After adjusting for conscientiousness (Model 2), the difference in the effect of extroversion between pandemic vs. pre-pandemic periods was only marginally significant (HR = 1.24/0.97 = 1.28, *p* ∼ 0.057, 95% CI 0.99–1.65). After adjusting for the other three personality traits (Model 3), the effect of extroversion prior to the pandemic was somewhat stronger, whereas the effect during the pandemic was somewhat weaker. Nonetheless, the association between extroversion and excess mortality remained unchanged (HR = 1.19/0.93 = 1.28, 95% CI 0.99–1.66).

**Fig. 1:**
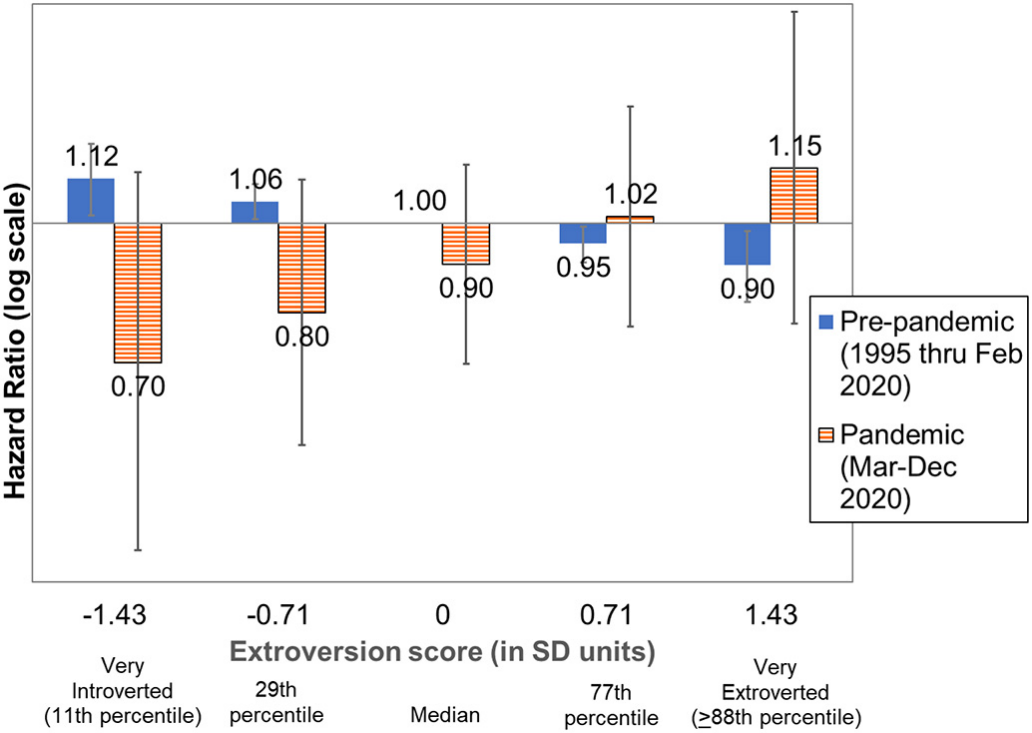
Fully-adjusted hazard ratios for mortality by level of extroversion and time period. Based on a Cox model that uses age as the time metric and adjusts for sex, race/ethnicity, the linear period trend in mortality decline (prior to the pandemic), the main effects for all 5 personality traits, a dichotomous indicator for the pandemic period (March–December 2020), and an interaction between the extroversion score and the pandemic indicator (Table S2, Model 3). The error bars represent the 95% confidence intervals. A substantial fraction (12%) of the sample scored the maximum value on extroversion (4, which is 1.43 SD above the mean); we defined this group as “very extroverted.” However, fewer than 0.1% of the sample scored the minimum value (1, which is 3.92 SD below the mean). To be more symmetric with the definition of “very extroverted” (i.e., those scoring at least 1.43 SD above the mean), we defined “very introverted” to represent those scoring 1.43 SD below the mean (which corresponds with the 11th percentile of the distribution). The other values show here were chosen to be as close as possible to the 25th, 50th, and 75th percentiles of the distribution.

To better demonstrate how the effect of extroversion changed during the pandemic, we computed the hazard ratios associated with selected levels of the extroversion score based on Model 3 (Fig. 1). Compared with someone who scored at the mean level of extroversion, mortality rates prior to the pandemic were 10% lower for a person who was very extroverted (i.e., maximum score, which comprised 12% of the sample), while the rates were 12% higher for someone who was very introverted (i.e., 11th percentile). However, the mortality advantage for more extroverted Americans disappeared during the COVID-19 pandemic. Although the differences are not significant (because of limited statistical power), the pattern of results suggests that, if anything, very extroverted individuals suffered *higher* mortality during the pandemic than those who were very introverted. Relative to those who scored at the mean level of extroversion, mortality rates during the pandemic appeared to be *highe*r for very extroverted individuals (HR = 1.15, 95% CI 0.77–1.72) and *lower* for those who were very introverted (HR = 0.70, 95% CI 0.43–1.14).

**Fig. 2:**
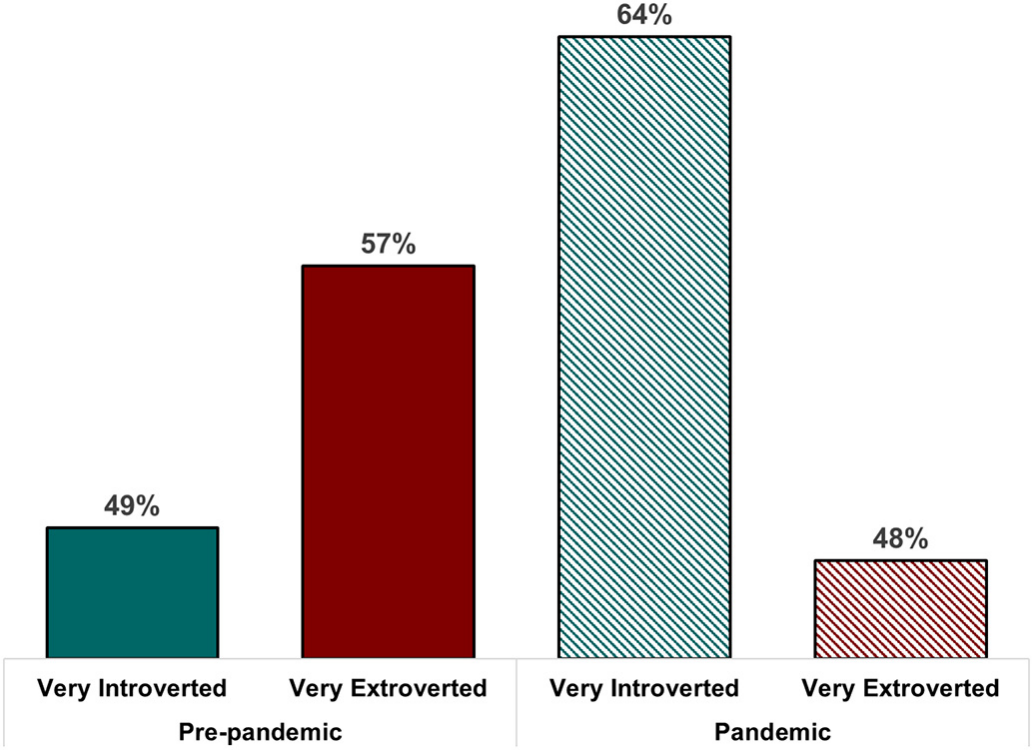
Estimated percentage surviving from age 25 to 85 by level of extroversion and time period. Estimates are based on Model 3 (Table S2) where the year is set to 2020, the dichotomous indicator for period is set to either pre-pandemic (January–February) or pandemic (March–December), and the extroversion score is fixed at the 11th percentile (i.e. scored 2.4 out of 4, which we defined as very introverted) or the top 12% of the distribution (i.e. scored 4 out of 4, which we defined as very extroverted). All other covariates (i.e. sex race/ethnicity and the other four personality traits) are fixed at the mean for the sample.

When we translated the estimated mortality rates into survival ratios before vs. during the pandemic (Fig. 2), we found that the percentage expected to survive from age 25 to 85 fell 9 percentage points for someone who was very extroverted (from 57% to 48%), whereas it increased 15 percentage points (from 49% to 64%) for their very introverted counterparts. Thus, survival of highly extroverted individuals during the pandemic was comparable to those who were highly introverted prior to the pandemic, whereas very introverted people had even better survival during the pandemic than highly extroverted individuals prior to the pandemic.

## 4 Discussion

As hypothesized, our results suggest that more extroverted people suffered higher excess mortality during the pandemic than their more introverted counterparts. We cannot say yet whether that pattern continued into 2021–22. The answer will have to wait until further mortality follow-up data become available.

Modern society is culturally biased toward extroverts, but a culture that favors extroversion and individualism is not the best prescription for surviving a pandemic. The mortality benefit of introversion during the pandemic was likely a result of reduced exposure to the risk of infection. Persons who were more introverted may have been more willing to limit social activities, practice social distancing, and avoid large social gatherings, which would have made them less prone to deaths resulting directly from COVID-19.

Some of the benefit may also derive from the ability of more introverted individuals to adapt more easily to reduced social interaction. If they were less likely than highly extroverted persons to succumb to depression, anxiety, and/or loneliness during the pandemic as previous studies suggest [[10], [11], [12], [13]], it could have suppressed mortality from other causes indirectly affected by the pandemic. If psychological distress contributed to excess mortality, we would expect to find an increase in suicide—the ultimate “death of despair” (a term commonly used to refer to deaths resulting from suicides and drug- and alcohol-related causes [20]). Yet, there is little evidence that suicide rates increased during the pandemic. In fact, contrary to many predictions, suicide mortality was significantly lower than expected throughout March–December 2020 [21], although there appears to have been a small increase among Americans aged 25–34 [22].

In contrast, other so-called “deaths of despair” increased dramatically in the US during the pandemic. Between 2019 and 2020, alcohol-related deaths increased 25% [23], while drug overdoses grew 30%, and in particular, deaths involving synthetic opioids such as fentanyl rose 55% [24]. If highly extroverted people were more likely than their more introverted counterparts to succumb to substance abuse—perhaps because of difficulty coping with the stressors imposed by the pandemic—it could have led to more excess mortality from external causes.

There are several limitations to this study, First, mortality during 2020 is almost certainly under-estimated. Deaths during 2020 were based on an early release file for the National Death Index (NDI), which according to the National Center for Health Statistics, accounted for only about 95% of all recorded US deaths in 2020 at the time of the NDI search [25]. Second, the MIDUS sampling frame excluded the institutionalized population, who suffered especially high mortality early during the early stages of the pandemic. Thus, mortality among the MIDUS cohort is likely to be lower than pandemic-related mortality for the population as a whole. Third, we have no information about the degree to which MIDUS participants complied with public health orders during the pandemic and whether it differed by personality. Nor do we have any information about self-destructive behaviors (e.g., alcohol and drug abuse) during the pandemic. Finally, the MIDUS sample under-represents minorities, who suffered higher mortality during the pandemic.

## 5 Conclusion

Our results suggest that the slight mortality advantage enjoyed by more extroverted people under normal circumstances disappeared during the first 10 months of the COVID-19 pandemic. Some would say that highly introverted people have been training for a pandemic their whole lives.

## Research data for this article

The original data used for this analysis are publicly available from ICPSR (https://www.icpsr.umich.edu/web/NACDA/series/203) or from the MIDUS portal (https://midus.colectica.org/). The data from Wave 1 of MIDUS can be downloaded from https://www.icpsr.umich.edu/web/NACDA/studies/2760. The most recent mortality followup for the original cohort can be downloaded from https://midus.colectica.org/item/midus.wisc.edu/0cf8bc9a-1daa-437b-9603-c02320a03fee.

## Funding

This work was supported by the 10.13039/100000049National Institute on Aging [grant numbers P01 AG020166, U19AG051426] and 10.13039/100005688the Graduate School of Arts and Sciences, 10.13039/100008064Georgetown University. The funders played no role in the study design; in the collection, analysis and interpretation of data; in the writing of the report; and in the decision to submit the article for publication.

## Declaration of Competing Interest

The authors declare that they have no known competing financial interests or personal relationships that could have appeared to influence the work reported in this paper.

## Appendix A Supplementary data

Supplementary material Image 1
