# Supplementary material for: In the midst of a pandemic, more introverted individuals may have a mortality advantage
Source: Dialogues Health. 2022 Nov 30;2:100087. doi: 10.1016/j.dialog.2022.100087 (PMC9708106; doi:10.1016/j.dialog.2022.100087)
Supplement: Supplementary file 1 — Supplementary material [file mmc1.docx]

Appendix A. Supplementary Data

In the midst of a pandemic, introverts may have a mortality advantage

Supplemental Methods

## Data

The Midlife in the United States (MIDUS) study targeted non-institutionalized, English-speaking adults aged 25–74^[[1]](#footnote-1)^ in the contiguous United States.^1^ At baseline (fielded January 1995–September 1996), national random digit dialing with oversampling of older people and men was used to select the main sample (*N*=3,487) and a sample of twin pairs (*N*=1,914). The study also included a random sample of siblings of individuals in the main sample (*N*=950) and oversamples from five metropolitan areas in the U.S. (*N*=757). The response rate for the phone interview ranged from 60% for the twin subsample to 70% for the main sample. Among those who completed the phone interview (*N*=7,108), 6,325 (89%) also completed mail-in self-administered questionnaires (SAQs).

## Measures

Vital status was ascertained through searches of the National Death Index (NDI), survey fieldwork, and longitudinal sample maintenance.^2^ The most recent mortality file for MIDUS includes deaths that occurred as late as December 2021, but the most recent NDI search covered the period through December 31, 2020; thus, mortality after 2020 is likely to be incomplete. The NDI search was based on final death data for 1995-2019 and an early release data file for 2020.

Measures for each of the “Big Five” personality traits were based on the degree to which the respondent endorsed a set of four to seven descriptors, using response categories that included “not at all” (1), “a little” (2), “some” (3), “a lot” (4).^3^ Extroversion was computed as the mean across five descriptors (Outgoing, Friendly, Lively, Active, Talkative; α=0.78). Conscientiousness was measured by 4 items (Organized, Responsible, Hardworking, Careless [reverse-coded]; α=0.56). Neuroticism included 4 items (Moody, Worrying, Nervous, Calm [reverse-coded]; α=0.75). Agreeableness was based on 5 items (Helpful, Warm, Caring, Softhearted, Sympathetic; α=0.81). Openness comprised 7 items (Creative, Imaginative, Intelligent, Curious, Broad-minded, Sophisticated, Adventurous; α=0.78).

Demographic confounders comprised sex, age, and race/ethnicity. Respondents were asked, “*What race do you consider yourself to be?*” We retained the first two response categories (i.e., Black and/or African American; White), but combined the remaining categories (i.e., Asian or Pacific Islander; multiracial; Native American or Aleutian Islander/Eskimo; other) into a group labeled “other race.” Ethnicity is based on reported countries of origin (“*Other than being American, what are your main ethnic origins? That is, what countries or continents are your ancestors from?*”). We classified respondents as Latina/o if they reported a country of origin in Mexico, Central America, Cuba, Dominican Republic, Puerto Rico, South America (including Brazil), or Spain. For respondents who were missing information regarding race/ethnicity from Wave 1 (2% of the sample), we used information from Wave 2: respondents were asked to identify the race with which they most closely identify (“*Which do you feel best describes your racial background? White, Black or African American, American Indian or Alaska Native, Asian, or Native Hawaiian or Pacific Islander?*”); Latina/o origin was also based on self-report (“*Are you of Spanish, or Hispanic or Latino descent, that is, Mexican, Mexican American, Chicano, Puerto Rican, Cuban or some other Spanish origin?*”).

## Statistical Analysis

Among the 6,325 respondents included in the analysis, the predictors with the highest percentage of missing data were race/ethnicity (2.9%) and personality measures (0.9-1.0%). We used the “ice” command in Stata 16.1 to perform multiple imputation. For the multiple imputation process, we used information for all the analysis variables as well measures of childhood socioeconomic status (SES), marital status, employment status, adult SES, smoking history, alcohol abuse, drug abuse, and physical limitations.

Descriptive statistics for all analysis variables are shown in Table S1. The mean score on extroversion was 3.2, with a distribution that was heavily weighted toward the extroverted end of the spectrum. Only 11% of the sample scored <2.4 (< 1.43 SD below the mean); 29% scored <2.8 (< 0.72 SD below the mean); 23% scored above 3.6 (> 0.72 SD above the mean); and 12% scored the maximum of 4 (1.42 SD above the mean). The mean score was highest for agreeableness (3.5) followed by conscientiousness (3.4) and lowest for neuroticism (2.2).

Age was treated as the “clock” in the Cox model, but we also included calendar year as a time-varying covariate to adjust for the period trend in mortality decline. Thus, we divided the survival history for each respondent into the intervals representing each calendar year from 1995 through 2019, the pre-pandemic portion of 2020 (i.e., January-February), and the final pandemic period (March-December 2020) in order to specify period as a time-varying covariate. We evaluated different specifications for the period trend (i.e., linear, quadratic, 5-year categories) based on mortality through 2019; the linear specification yielded the best fit (i.e., based on the Bayesian Information Criterion). Thus, we treated period as linear in the final models.

Table S1. Descriptive statistics for analysis variables, *N*=6,325^a^

| Variable | Mean (SD) or N (%) |
| --- | --- |
| Age at baseline^b^ (20-75), mean (SD)^b^ | 46.9 (12.9) |
| Male, N (%) | 3004 (47.5) |
| Non-Latina/o White, N (%) | 5582 (88.3) |
| Non-Latina/o Black, N (%) | 328 (5.2) |
| Non-Latina/o Other race, N (%) | 243 (2.7) |
| Latina/o, N (%) | 172 (3.8) |
| Extroversion (1-4), mean (SD) | 3.2 (0.6) |
| Conscientiousness (1-4), mean (SD) | 3.4 (0.4) |
| Neuroticism (1-4), mean (SD) | 2.2 (0.7) |
| Openness (1-4), mean (SD) | 3.0 (0.5) |
| Agreeableness (1-4), mean (SD) | 3.5 (0.5) |
| Died by 12/31/2020, N (%) | 1767 (27.9) |

^a^ With the exception of mortality, all variables are measured at baseline (1995-96).

^b^ At the end of mortality follow-up (12/31/2020), survivors were aged 31-98.

Table S2. Hazard Ratios (95% CIs) from Models Predicting Age-Specific Mortality, MIDUS, 1995-2020

|  | (1) | (2) | (3) |
| --- | --- | --- | --- |
| Male | 1.34*** | 1.32*** | 1.37*** |
|  | (1.22 - 1.48) | (1.20 - 1.45) | (1.25 - 1.52) |
| Non-Latina/o White | 1.00 | 1.00 | 1.00 |
| Non-Latina/o Black | 1.39** | 1.38** | 1.39** |
|  | (1.11 - 1.74) | (1.10 - 1.73) | (1.11 - 1.75) |
| Non-Latina/o Other race | 0.94 | 0.87 | 0.87 |
|  | (0.67 - 1.33) | (0.61 - 1.25) | (0.61 - 1.24) |
| Latina/o | 0.99 | 0.99 | 0.98 |
|  | (0.73 - 1.35) | (0.73 - 1.34) | (0.72 - 1.33) |
| Year – 1995^b^ | 0.99*** | 0.99*** | 0.99*** |
|  | (0.98 - 0.99) | (0.98 - 0.99) | (0.98 - 0.99) |
| Pandemic (Mar-Dec 2020)^c^ | 0.90 | 0.90 | 0.90 |
|  | (0.70 - 1.16) | (0.70 - 1.16) | (0.70 - 1.16) |
| Extroversion^a^ during: |  |  |  |
| Pre-pandemic^d^ | 0.93** | 0.97 | 0.93* |
|  | (0.88 - 0.97) | (0.92 - 1.02) | (0.87 - 0.99) |
| Pandemic^e^ | 1.20 | 1.24 | 1.19 |
|  | (0.93 - 1.54) | (0.96 - 1.59) | (0.92 - 1.53) |
| Conscientiousness^a^ |  | 0.86*** | 0.86*** |
|  |  | (0.82 - 0.91) | (0.82 - 0.91) |
| Neuroticism |  |  | 1.09** |
|  |  |  | (1.03 - 1.15) |
| Openness^a^ |  |  | 1.04 |
|  |  |  | (0.98 - 1.10) |
| Agreeableness^a^ |  |  | 1.07* |
|  |  |  | (1.01 - 1.13) |

* p<0.05, ** p<0.01, *** p<0.001

^a^ Standardized; the hazard ratio (HR) represents the effect per SD.

^b^ Represents the per year change in mortality relative to 1995.

^c^ Represents the difference in mortality during the pandemic (Mar-Dec 2020) compared with the pre-pandemic period (1995 through Feb 2020) after adjusting for period mortality decline.

^d^ Represents the effect of extroversion (per SD) on the mortality rate prior to the pandemic. We have reparameterized this model to include effects for extroversion during the pre-pandemic and during the pandemic periods rather than a main effect for extroversion and an interaction effect. This HR is the same as the main effect in the standard model specified with a main and interaction effect.

^e^ Represents the effect of extroversion (per SD) on the mortality rate during the pandemic. This HR equals the product of the HRs for the main and interaction effects in a standard specification (i.e., the exponentiated sum of the coefficients). To obtain the HR for the interaction effect in the standard specification (i.e., the effect of extroversion on the level of excess mortality; that is, the degree to which the effect of extroversion differed between the pre-pandemic and pandemic periods), one can divide this hazard ratio by the corresponding hazard ratio prior to the pandemic.

## References

1. Brim OG, Baltes PB, Bumpass LL, et al. National Survey of Midlife Development in the United States (MIDUS 1), 1995-1996: Description of MIDUS Samples. Inter-university Consortium for Political and Social Research [distributor], Version 19. Published 2020. Accessed July 28, 2021. https://doi.org/10.3886/ICPSR02760.v19

2. Ryff C, Almeida D, Ayanian J, et al. Documentation of mortality statistics and cause of death codes for core (non-refresher) MIDUS and Milwaukee samples. Published 2022. Accessed March 17, 2022. https://midus-study.github.io/public-documentation/Mortality/Core/MIDUS_Core_DocumentationOfMortality_20220316.pdf

3. Lachman ME, Weaver SL. *The Midlife Development Inventory (MIDI) Personality Scales: Scale Construction and Scoring. Technical Report*.; 1997:9. https://www.brandeis.edu/psychology/lachman/pdfs/midi-personality-scales.pdf

1. Although the sampling frame targeted adults aged 25-74, the final sample included a few respondents aged 20-24 (*N*=15) or aged 75 (*N*=4) at the baseline phone interview. [↑](#footnote-ref-1)
